# Double-Strand Break Repair and Holliday Junction Processing Are Required for Chromosome Processing in Stationary-Phase *Escherichia coli* Cells

**DOI:** 10.1534/g3.111.001057

**Published:** 2011-11-01

**Authors:** Ashley B. Williams, Kyle M. Hetrick, Patricia L. Foster

**Affiliations:** * Molecular and Computational Biology, University of Southern California, Los Angeles, California 90089; † Department of Biology, Indiana University, Bloomington, Indiana 47405

**Keywords:** DNA repair, stationary phase, homologous recombination, genome stability, double-strand break repair

## Abstract

As nutrients are depleted and cell division ceases in batch cultures of bacteria, active processes are required to ensure that each cell has a complete copy of its genome. How chromosome number is manipulated and maintained in nondividing bacterial cells is not fully understood. Using flow cytometric analysis of cells from different growth phases, we show that the Holliday junction–processing enzymes RuvABC and RecG, as well as RecBCD, the enzyme complex that initiates DNA double-strand break repair, are required to establish the normal distribution of fluorescent peaks, which is commonly accepted to reflect the distribution of chromosome numbers. Our results reveal that these proteins are required for the proper processing of chromosomes in stationary phase.

The molecular processes by which bacterial cells replicate, repair, recombine, and partition their chromosomes during exponential growth have been extensively studied, but little is known about how DNA is manipulated during stationary phase when cells are not dividing. It is usually assumed that DNA metabolism ceases when cell division stops; however, at least three reports have documented that tritium-labeled thymidine can be incorporated into the genomic DNA of nondividing *Escherichia coli* cells (Boe 1990; Grivell *et al.* 1975; Tang *et al.* 1979). Evidence that DNA synthesis and recombination continue in stationary-phase cells is also provided by a phenomenon known as “adaptive mutation,” during which mutations occur in the absence of cell division. In the most-studied case, reversion to lactose utilization of *Escherichia coli* strain FC40, adaptive mutation requires recombination functions, proteins for double-strand break repair, and the DNA damage-induced DNA polymerase IV (Pol IV) (reviewed in Foster 2007). Taken together, these results suggest that DNA-related molecular processes continue into stationary phase, even in the absence of bulk chromosomal replication.

As cells enter stationary phase, a complex cascade of events prepares them to survive famine conditions. Genes required for exponential growth are downregulated, and genes required for survival during starvation conditions are upregulated (Patten *et al.* 2004; Rahman *et al.* 2006). The chromosome is also reorganized and compacted, which can contribute to gene regulation but also protects the DNA from environmental and endogenous DNA-damaging agents [for examples, see Frenkiel-Krispin *et al.* (2004) and Nair and Finkel (2004)]. These changes in chromosome organization are largely due to several DNA binding proteins, some of which are upregulated in stationary-phase cells (reviewed in Dorman 2009). Interestingly, during the same period that this chromosomal reorganization occurs, transcription of the *dnaN* and *recF* genes, which are adjacent in a complex operon, is activated from promoters that are not used during exponential growth (Villarroya *et al.* 1998). *dnaN* encodes the replication processivity factor β, and *recF* encodes a protein involved in recombinational DNA repair. The increased expression of these proteins as cells enter stationary phase suggests that recombination processes continue to be important after genomic replication has ceased.

Holliday junctions (HJ) are mobile four-stranded DNA structures that are formed during homologous recombination. To restore two intact double-stranded DNA molecules, HJs must be processed and resolved. *E. coli* has two pathways for processing HJs: the RuvABC resolvasome and the RecG translocase. RuvA and RuvB bind to HJs, hydrolyze ATP, and migrate the junction along the DNA. An interaction with RuvA stimulates RuvC to cleave the DNA, resolving the junction (reviewed in West 1997). RecG can also bind and migrate HJs; however, unlike the RuvABC resolvasome, RecG does not have an intrinsic ability to resolve the structure (Mcglynn and Lloyd 1999). While a need for HJ processing in stationary-phase cells has not been explicitly shown, RuvABC promotes and RecG inhibits stationary-phase adaptive mutation (Foster *et al.* 1996; Harris *et al.* 1996; Williams and Foster 2007).

During normal cell growth, double-strand breaks can be caused by reactive oxygen species formed by normal cellular metabolism or failure of replication forks (for example, see Michel *et al.* 2007). Repair of DNA double-strand breaks via the RecBCD pathway leads to the formation of Holliday junctions. When a double-strand break occurs, the RecBCD complex binds to and processes the double-strand end, producing a 3′ single-strand overhang. RecA then catalyzes invasion of this strand into a homologous duplex, ultimately leading to the formation of one or two HJs (reviewed in Dillingham and Kowalczykowski 2008). RecBCD is also required for stationary-phase adaptive mutation (Harris *et al.* 1994), but to date, no other requirement for RecBCD in stationary-phase cells has been reported.

Flow cytometric analysis of cell populations in batch cultures revealed that stationary-phase cells might contain one, two, four, or even eight chromosomes (Åkerlund *et al.* 1995; Boye and Løbner-Olesen 1991). Because DNA recombination activities are required for stationary-phase adaptive mutation, we sought to determine whether these activities also function in the establishment and/or maintenance of higher-order genomic structures. Specifically, we examined whether the RuvABC resolvasome, the RecG HJ translocase, and the RecBCD double-strand break repair machinery are required to generate the distribution of chromosome numbers that normally arises during early- to mid-stationary phase. The results presented here reveal that RuvABC, RecG, and RecBCD ensure proper chromosome processing during stationary phase.

## Materials and Methods

### Bacterial strains

All bacterial strains used in this study are *Escherichia coli* K-12 derivatives and are described in Table 1.

**Table 1:** Strains used in this study

Strain	Genotype	Reference
FC36	F^−^ *ara* Δ(*gpt-lac*)5 *thi* Rif^R^	(Cairns and Foster 1991)
FC400	FC36 *recB21 argA*::Tn*10*	(Foster *et al.* 1996)
FC457	FC36 *recG258*::*d*Tn*10*Kan	(Foster *et al.* 1996)
FC484	FC36 *ruvA60*::Tn*10*	(Foster *et al.* 1996)
FC513	FC36 *ruvA60*::Tn*10 recG258*::*d*Tn*10*Kan	(Foster *et al.* 1996)

### Media and growth conditions

Cells of each strain were cultured in Luria-Bertani (LB) broth (Miller 1992) at 37° with aeration. After growth overnight, cultures were diluted 1:1000 into 25 mL fresh LB broth with no antibiotics in 125 mL flasks and grown at 37° with shaking. The optical density (OD_600_) was measured at each time point by removing appropriate samples of the cultures. In a separate experiment, samples were taken at comparable time points, diluted appropriately, and plated on LB 15% agar medium to determine the total number of colony-forming units (CFU).

### Analysis of cellular DNA content by flow cytometry

At each time point, 0.5 mL samples were removed from the 25 mL LB broth cultures and added to 4.5 mL of 78% ice-cold ethanol to produce a final concentration of 70% ethanol. The fixed cells were stored at −20° until analysis. For analysis, the cells were first rinsed twice with 10 mL of 1X phosphate buffered saline (PBS) at 4°, then stained with propidium iodide in a solution of 0.2 mg/mL RNase (Sigma, R4875), 0.01% Triton X-100 (Thermo Fisher Scientific, Inc., Waltham, MA), and 3 μm propidium iodide (Sigma, P4864) in 1X PBS.

DNA content was analyzed using a FACSCalibur flow cytometer (BD Biosciences, San Jose, CA). Approximately 50,000 cells were analyzed at a speed of 300 to 900 cells per second. The FL2-width (FL2-W) indicates relative cell size, and FL2-area (FL2-A) indicates total propidium iodide fluorescence. To examine how cell size might affect the fluorescence profiles, data analyses including different cell populations (gatings) were done. The overall fluorescence profiles were unaffected by these different gatings, so the histograms shown in Figures 3–7 and supporting information, Figure S1, Figure S2, Figure S3, Figure S4, and Figure S5 represent the entire data set for each sample. Data analysis was performed using the FlowJo Version 8 for Mac (Tree Star, Inc., Ashland, OR).

### Measurement of DNA synthesis

Overnight cultures were diluted by a factor of 10^5^ into 50 mL fresh LB broth in 125 mL flasks and grown at 37° with shaking. The OD_600_ was monitored throughout the experiment. Samples were collected for CFU determination and 5-ethynyl-2′-deoxyuridine (EdU) labeling at 6.5–8.5 hr (exponential phase; OD_600_ = 0.6 ± 0.1), 8.5–11 hr (stationary phase; OD_600_ = 1.5 ± 0.1), and 21 hr (late-stationary phase; OD_600_ = 1.8 ± 0.04) after inoculation. The times of sampling differed from strain to strain depending on growth rate. Cells were labeled with EdU using the Click-iT EdU Alexa Fluor 488 Azide Imaging Kit (Molecular Probes, Eugene, OR; #C10337) as in Ferullo *et al.* 2009. Briefly, 2 mL aliquots of culture were added to tubes with 24 μL of 10 mm EdU and incubated for 15 min at 37° with shaking. Cells were fixed with 90% methanol and stored at 4° for 3–8 days. Repeated experiments confirmed that storage time did not affect results. For analysis, cells were washed with PBS, permeabilized in a 0.5% Triton X-100/PBS solution for 30 min at room temperature, washed again with PBS, suspended in Click-iT reaction mixture, and incubated in the dark for 30 min at room temperature. Cells were then washed with reaction rinse buffer and suspended in PBS. Fluorescence microscopy was performed using a Nikon 80i microscope with a FITC HYQ filter (Nikon Instruments, Inc., Melville, NY). Phase-contrast and fluorescence images were viewed and captured as described below. The number of CFUs was determined as described above.

### Phase-contrast microscopy

Phase-contrast microscopy of unfixed cells was performed using a Nikon 80i microscope (Nikon Instruments). The phase images were viewed using a Nikon Plan Apo 100X objective (Nikon Instruments), and the images were captured using a Photometrics Coolsnap HQ2 camera (Photometrics, Tuscon, AZ) and Metamorph image software (Molecular Devices, Sunnyvale, CA). Representative fields of view are shown in the figures.

## Results

### Growth rates of recombination-defective mutant strains

**Figure 1:**
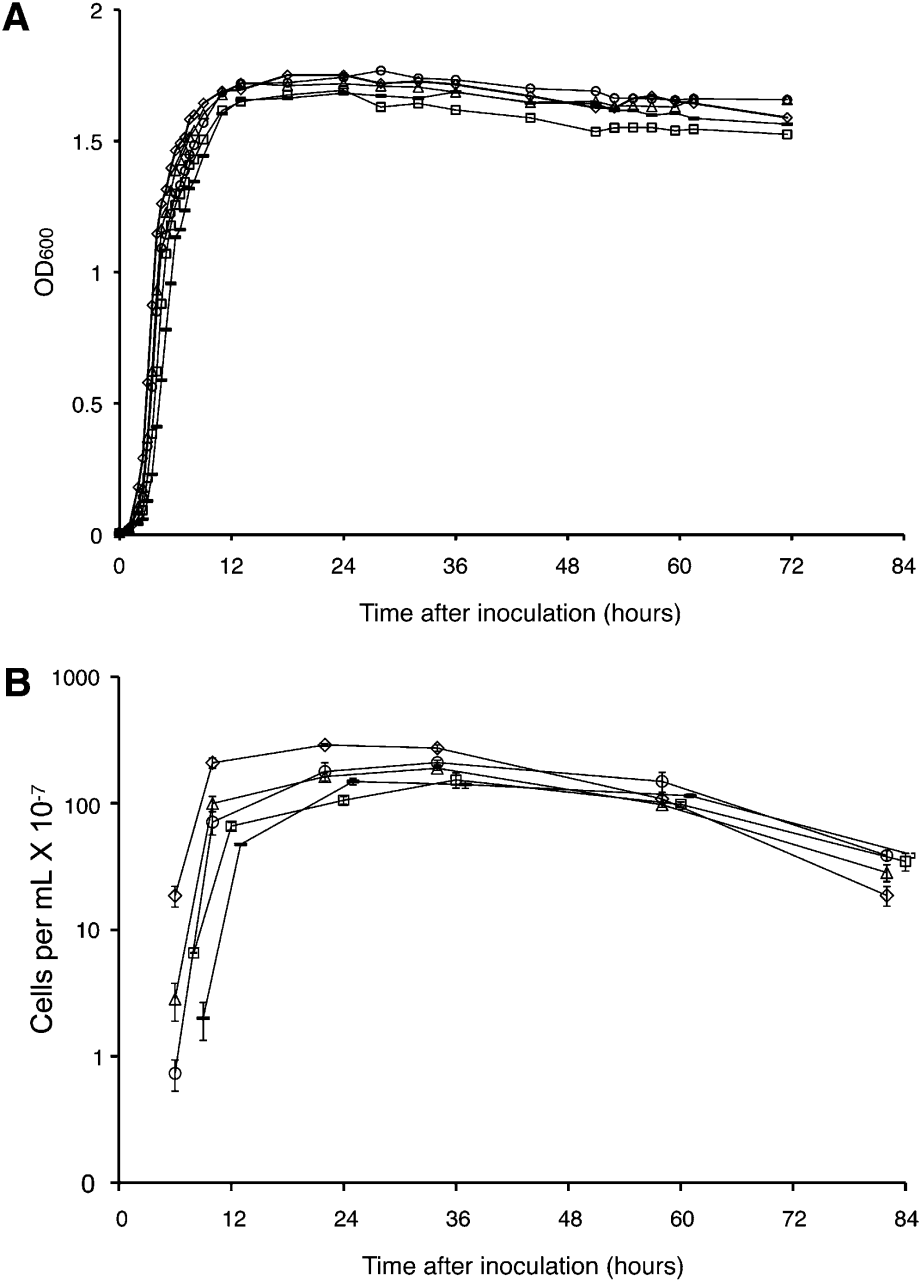
Growth parameters and viability of wild-type and recombination-defective strains over time. (A) Optical densities measured at a wavelength of 600 nm (OD_600_) are plotted against the time (in hours) after inoculation. Samples were removed from the cultures at each time point marked. (B) At each time point, samples were collected from cultures started by diluting overnight cultures by a factor of 10^5^ into fresh LB medium; the number of colony-forming units was determined by appropriate dilution and plating on LB agar. The number of cells per milliliter is plotted on a logarithmic scale against the time (in hours) after inoculation. Diamonds = FC36 (wild-type); triangles = FC457 (*recG258*); circles = FC484 (*ruvA60*); squares = FC400 (*recB21*); dashes = FC513 (*recG258 ruvA60*).

As shown in Figure 1A, the growth rates of the *recG258*, *ruvA60*, *recB21*, and *recG258 ruvA60* mutant strains differed little from that of the wild-type strain. Based on a least-squared fit of the linear parts of the curves in Figure 1A, the growth rates of the mutant strains differed from that of the wild-type strain by no more than 15%. All the mutant strains had somewhat longer lag phases than the wild-type strain; the longest was the lag phase of the *recG258 ruvA60* double-mutant strain, which was about twice that of the wild-type strain. All of the strains reached comparable final optical densities. In the discussions below, we consider the 7–9 hr time period to be the entry into stationary phase and all times after 11 hr to be stationary phase. As measured by CFUs, after about 40 hr, the culturable cell number began to slowly decline, so that by 80 hr after inoculation only 10–30% of the cells could produce colonies on LB agar (Figure 1B).

### Changes in cell size of recombination-defective strains

**Figure 2:**
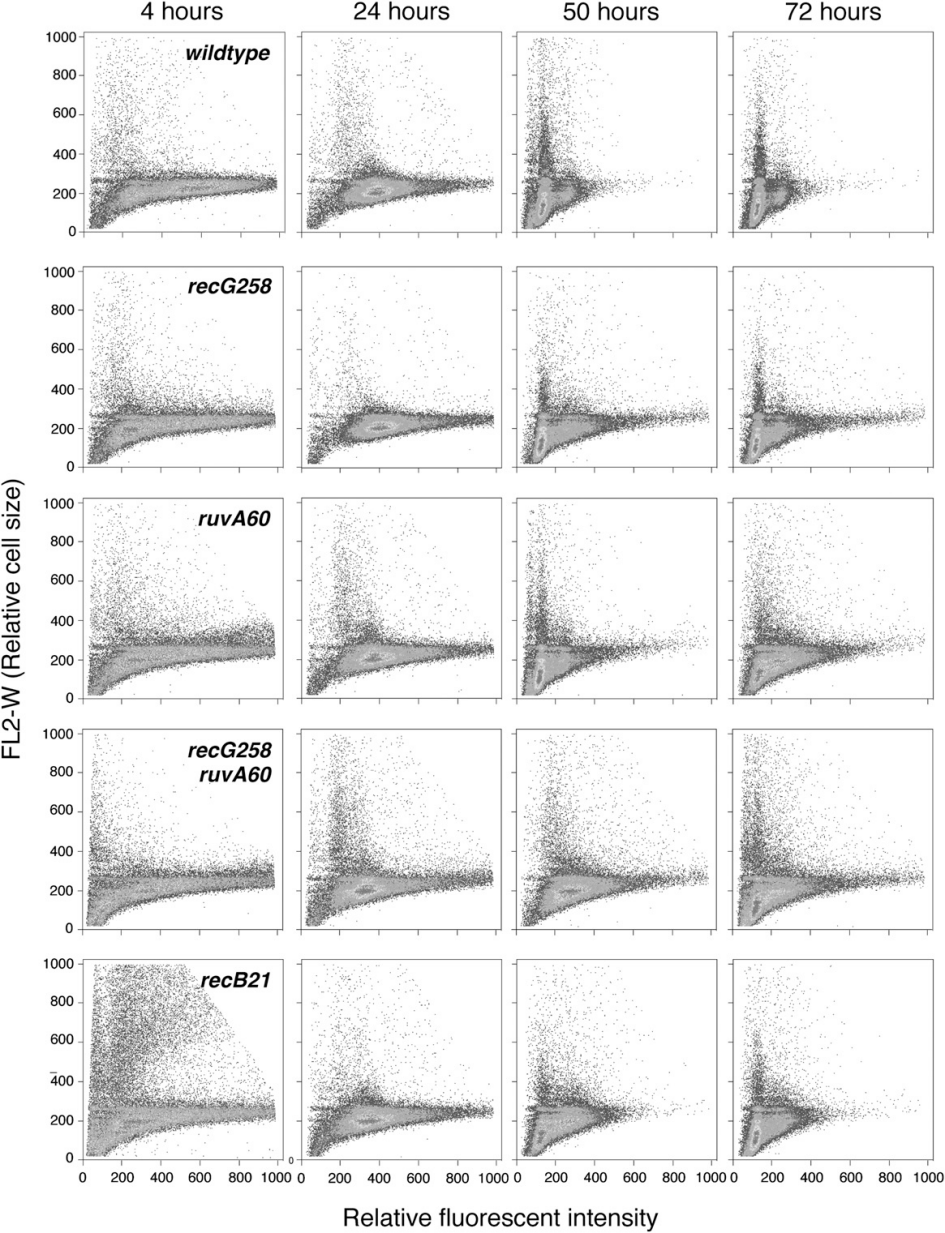
Effects of recombination defects on cell size. The relative FL2-W values (cell size) are plotted against the relative FL2-A values (total propidium iodide fluorescence) in arbitrary units. Increasing values on the Y-axis indicate larger cells, and increasing values on the X-axis indicate greater total propidium iodide fluorescence intensity.

**Figure 3:**
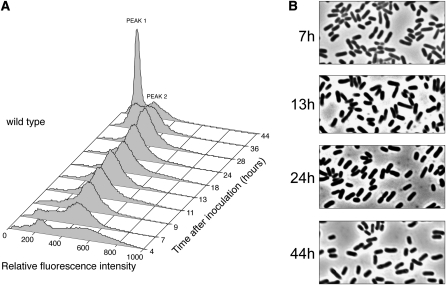
Populations of cells with distinct fluorescence intensities develop during growth and stationary phase of the wild-type strain. Propidium iodide–stained cells of the wild-type strain (strain FC36) were analyzed by flow cytometry at the indicated time points. (A) The numbers of cells (Y-axis) are plotted against their relative fluorescence intensities (X-axis). The histograms from the time points indicated are aligned along the Z-axis. (B) Phase-contrast micrographs of unfixed cells from the time points indicated (1000× magnification).

**Figure 4:**
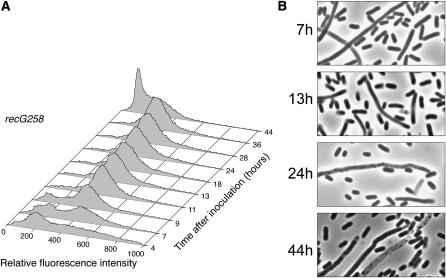
The Holliday junction–migrating protein RecG is required for the development of normal fluorescent population dynamics during stationary phase. Propidium iodide–stained cells (strain FC457) were analyzed by flow cytometry at the indicated time points. (A) The numbers of cells (Y-axis) are plotted against their relative fluorescence intensities (X-axis). The histograms from the time points indicated are aligned along the Z-axis. (B) Phase-contrast micrographs of unfixed cells from the time points indicated (1000× magnification).

**Figure 5:**
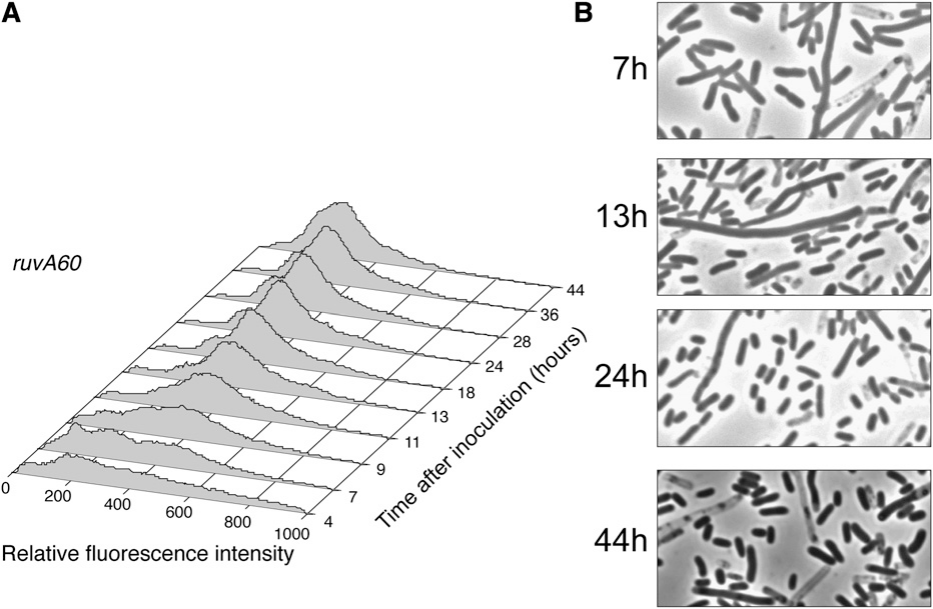
The Holliday junction–processing RuvABC complex is required for normal fluorescent population dynamics during growth and in stationary phase. Propidium iodide–stained cells (strain FC484) were analyzed by flow cytometry at the indicated time points. (A) The numbers of cells (Y-axis) are plotted against their relative fluorescence intensities (X-axis). The histograms from the time points indicated are aligned along the Z-axis. (B) Phase-contrast micrographs of unfixed cells from the time points indicated (1000× magnification).

**Figure 6:**
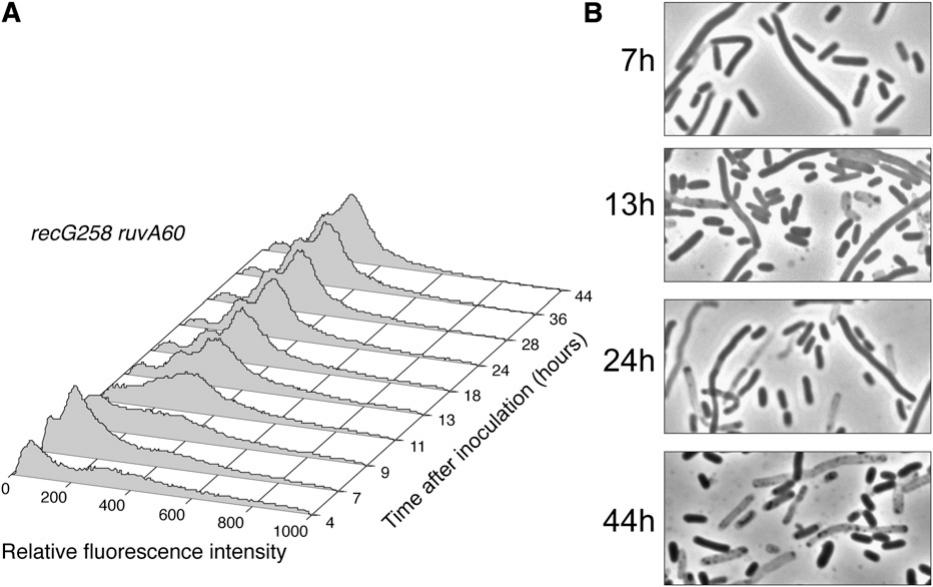
Loss of both Holliday junction–processing pathways severely alters fluorescent population dynamics during growth and in stationary phase. Propidium iodide–stained cells (strain FC513) were analyzed by flow cytometry at the indicated time points. (A) The numbers of cells (Y-axis) are plotted against their relative fluorescence intensities (X-axis). The histograms from the time points indicated are aligned along the Z-axis. (B) Phase-contrast micrographs of unfixed cells from the time points indicated (1000× magnification).

**Figure 7:**
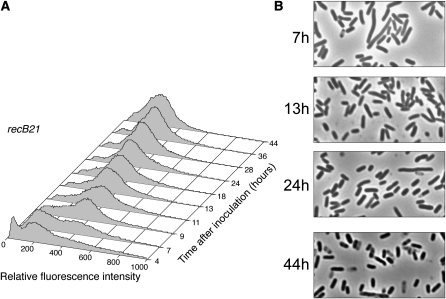
Loss of RecB alters fluorescent population dynamics during growth and in stationary phase. Propidium iodide–stained cells (strain FC400) were analyzed by flow cytometry at the indicated time points. (A) The numbers of cells (Y-axis) are plotted against their relative fluorescence intensities (X-axis). The histograms from the time points indicated are aligned along the Z-axis. (B) Phase-contrast micrographs of unfixed cells from the time points indicated (1000× magnification).

Loss of recombination functions can induce the SOS response, leading to cell filamentation (Chua *et al.* 1993; Lloyd and Buckman 1991; Mccool *et al.* 2004; Witkin 1976). We used flow cytometric analysis of fixed and propidium iodide–stained cells to examine the morphological heterogeneity of the wild-type (FC36) and isogenic mutant strains during the course of our experiments. Approximately 50,000 cells of each strain were analyzed, and each cell is represented by a dot in the plots shown in Figure 2. The magnitude of the FL2-W parameter is determined by the amount of time each cell remained in the path of the laser beam and is thus a measure of cell size. In each panel of Figure 2, the FL2-W value of each cell (vertical axis) is plotted against its relative fluorescence intensity (horizontal axis). Plots from several time points during the course of incubation are presented. As the plots show, the great majority of the cells in all the cultures were approximately the same size as the wild-type cells, but the cultures of the mutant strains also contained larger, presumably filamenting cells. With the exception of the 4 hr time points, the distributions of cell sizes of the *recG258* and *recB21* mutant strains were most similar to that of the wild-type strain, whereas the *ruvA60* and *recG258 ruvA60* mutant strains showed the greatest increase in the proportion of larger cells. These results were confirmed by the phase-contrast images in Figures 3–7 and Figure S1, Figure S2, Figure S3, Figure S4, and Figure S5, which show that the morphology of the wild-type strain was constant throughout the course of the experiment but that the cultures of the mutant strains contained both normal and filamenting cells. The reason for the large variation in cell size in the *recB21* mutant strain 4 hr after inoculation is not clear, but it could be due to double-strand breaks that formed in the stationary-phase cells that were used as the inoculum; as the cells began to divide, these breaks would then induce the SOS response and filamentation. Nonetheless, even in the strains mutant for double-strand break repair, HJ processing, or both, the majority of cells were of normal size and morphology during the course of these experiments. In Figures 3–7 and Figure S1, Figure S2, Figure S3, Figure S4, and Figure S5 presenting flow cytometry data, the histograms include the entire cell populations, as omission of the larger cells had no noticeable impact on the final plots.

### Development of the wild-type pattern of fluorescence intensities during exponential and early- to mid-stationary phase requires HJ processing

As shown in Figure 3A, as wild-type cells progressed through stationary phase, populations of cells with different fluorescence intensities developed. Previous studies have identified the two separate fluorescent peaks that develop in LB-grown cultures early in stationary phase to consist of cells containing two (lower fluorescence intensity) or four (higher fluorescence intensity) chromosomes (Åkerlund *et al.* 1995). For the remainder of this article, the low fluorescence intensity peak is referred to as peak 1, and the higher fluorescence intensity peak is referred to as peak 2. In our experiments, the wild-type strain contained predominantly cells in peak 2 until 28 hr postinoculation, and then a population of cells in peak 1 developed between 28 and 44 hr postinoculation. The fluorescence profile of our strain at 44 hr postinoculation was similar to that obtained at 28 hr postinoculation by Åkerlund and Colleagues (1995), except our cultures had a higher proportion of cells in peak 1. In addition, we did not see the small peak at even higher intensity that Åkerlund *et al.* (1995) identified as cells with eight chromosomes. These differences are probably due to media composition: we used LB whereas Åkerlund *et al.* (1995) used LB plus glucose. The phase-contrast images in Figure 3B show that up to 44 hr postinoculation, the morphology of wild-type cells did not vary noticeably.

As shown in Figure 4A, the fluorescence profiles of a strain defective for the RecG translocase were similar to those of the wild-type strain during growth and early-stationary phase. However, the *recG* mutant strain was slow to develop the large peak 1 cell population that was present in the wild-type population by 36 hr postinoculation. Figure 4B shows that some filamentous cells were present in the culture of the *recG258* mutant strain; however, cells of normal size and shape dominated the culture at all time points sampled.

To further examine the role of HJ processing, we analyzed a strain carrying the *ruvA60* mutant allele that inactivates the RuvABC HJ resolvasome. As shown in Figure 5A, the fluorescence profiles of the *ruvA60* mutant strain were similar to those of the wild-type strain up to 28 hr postinoculation; however, the *ruvA60* mutant strain failed to develop the peak 1 population by 44 hr postinoculation. The phase-contrast images in Figure 5B show that, like the *recG258* mutant strain, the population of the *ruvA60* mutant strain contained filamenting cells, although again, most cells were of normal size and shape.

In a *recG258 ruvA60* double-mutant strain, HJs cannot be processed. As shown in Figure 6A, the distributions of fluorescence intensities at all time points for this strain were very different from the wild-type strain. A peak 1 population appeared during exponential phase (as well as what appears to be a peak of even lower intensity that could correspond to cells with one chromosome), briefly disappeared as the cells entered stationary phase, and then reappeared at 13 hr postinoculation, a time earlier than when a similar population appeared in the wild-type or the single mutant strain. However, the peak 1 population did not become dominant, even in late-stationary phase, suggesting that HJ processing is required for cells to enter this population. The morphologies of cells are shown at sample time points in Figure 6B.

### Development of the wild-type pattern of fluorescence intensities during exponential and early- to mid-stationary phase requires the enzyme that initiates double-strand break repair

HJs are formed during repair of double-strand breaks by the RecBCD complex. To test whether RecBCD was required for changes in fluorescence, we analyzed the distributions of fluorescence intensities in the population of the *recB21* mutant strain at various points during incubation. As shown in Figure 7A, the fluorescence profiles of the *recB21* mutant strain were very similar to those of the *recG258 ruvA60* double-mutant strain: peak 1 appeared during exponential phase, briefly disappeared as the cells entered stationary phase, and then reappeared but never became dominant. This result suggests that double-strand break repair is required for the development of the peak 1 population observed in wild-type cultures. As shown in Figure 7B, few filamentous cells were observed in the *recB21* culture in stationary phase. The lack of filamentation indicates that the SOS response was not induced in this strain to the extent that it was in the other mutant strains tested, a result previously reported by Chua *et al.* (1993). This could be because the RecA loading activity of RecBCD is required to produce the SOS-inducing signal, and this function cannot be performed by the alternative double-strand break repair enzyme RecFOR (Ivančić-Baće *et al.* 2006).

### Changes in fluorescence populations continue late into stationary phase

The fluorescence profiles of all of the strains tested were dynamic even to 44 hr postinoculation or approximately 30 hr after the onset of stationary phase. To determine whether further changes in the population distributions occurred, we continued the analysis to 72 hr postinoculation, although these results should be considered with caution as a large proportion of the cells in each culture were not culturable at these late time periods (see Figure 1B). The fluorescence profile of the wild-type strain was stable from 44 hr to 72 hr postinoculation, and that of the *recG258* mutant strain was similar to wild-type but slightly slower to stabilize (see Figure S1 and Figure S2). In each of the other mutant strains, a large population of cells in peak 1 eventually developed, but these peaks developed slowly, particularly in the *ruvA60 recG258* double-mutant strain (see Figure S3, Figure S4, and Figure S5).

### Ongoing DNA replication does not explain variations in fluorescence intensity

To determine whether ongoing DNA synthesis in the recombination mutant strains could explain our results, we tested whether these strains incorporate EdU, a thymidine analog, into their DNA during stationary phase. Incorporated EdU can be detected by a cycloaddition reaction with a fluorochrome-tagged azide, yielding a fluorescent product (Ferullo *et al.* 2009; Salic and Mitchison 2008). At the appropriate growth stage, cells were incubated briefly with EdU to allow incorporation; they were then fixed, reacted with the Click-iT reagent, and imaged using fluorescence microscopy.

Images of cells in exponential phase revealed that greater than 90% of the cells of the wild-type, *recG*, *recB*, and *ruvA* mutant strains contained foci, whereas only 74% of the cells of the *ruvA recG* double-mutant strain contained foci (Table 2 and Figure S6). These results indicated that, as expected, most exponential-phase cells were synthesizing DNA, although fewer were in strains defective in double-strand break repair and HJ processing.

**Table 2:** Proportions of cells with foci in exponential, stationary, and late-stationary phases

	Exponential Phase		Stationary Phase		Late-Stationary Phase	
	Harvested 6.5–8.5 hr		Harvested 8.5–11 hr		Harvested 21 hr	
	% of cells with foci	*N*	% of cells with foci	*N*	% of cells with foci	*N*
Wild-type	99	1697	17	3174	0	808
*recB21*	91	538	17	2963	0	454
*recG258*	98	1019	18	633	0	262
*ruvA60*	91	620	2	1370	0	379
*recG258 ruvA60*	74	1392	1	1491	0	327

Cultures were grown, exposed to EdU, harvested, and visualized as described in *Materials and Methods*. “Harvested” refers to the time (in hours) after inoculation that cells were collected and fixed. These times are greater than the harvesting times for the flow cytometry samples because the cultures were started with smaller inocula. For each sample taken from the exponential phase and stationary phase, three fields were imaged, and the total number of cells with fluorescent foci was counted. For each sample taken in late-stationary phase, three fields were imaged, but the total number of cells in only one field was counted. Wild-type = FC36; *recB21*= FC400; *recG258* = FC457; *ruvA60* = FC484; *recG258 ruvA60* = FC513.

Images of cells in early-stationary phase revealed substantial decreases in the numbers of cells that could incorporate EdU (Table 2). Less than 20% of the cells of the wild-type, *recB*, and *recG* mutant strains contained foci, and only 1–2% of the cells of the *ruvA* mutant and *ruvA recG* double-mutant strains contained foci. These results indicate that DNA synthesis was mostly completed by early-stationary phase.

No fluorescent foci were detected in any cells exposed to EdU after about 12 hr into stationary phase, even though nearly all of the cells were culturable (Figure 1B). These results indicate that DNA synthesis, at least at the level required for detection by EdU incorporation, had ceased in all cells by this time. An alternative is that cells in late-stationary phase cannot incorporate EdU; however, this explanation is unlikely as previous work has shown that such cells can incorporate tritium-labeled thymidine (Tang *et al.* 1979).

While the most severe abnormalities in DNA content in stationary-phase cells, as detected by fluorescence profiles, were seen in the *recB21* and *recG ruvA* mutant strains, these strains had different amounts of EdU incorporation in stationary phase (17% and 1%, respectively). Thus, it is unlikely that ongoing DNA synthesis in stationary phase explains the differences in fluorescence profiles that we observed among the mutant populations.

## Discussion

The data described in this study reveal new details about how *E. coli* cells maintain their genomic organization during various phases of growth. Using flow cytometric analysis, we showed that in cultures of wild-type cells, two primary cell populations with different amounts of DNA develop as the cells enter stationary phase (peak 1 and peak 2 populations); these populations correspond to those previously reported to contain two and four chromosomes (Åkerlund *et al.* 1995). In our experiments, these distinct populations did not develop in cultures of the wild-type strain until 36–44 hr postinoculation, which was 18–26 hr after the onset of stationary phase. These times are several hours later than reported by Åkerlund *et al.* (1995), most likely because our cultures were grown in the commonly used LB medium, whereas in Åkerlund *et al.* (1995), the cultures were grown in the richer LB plus glucose medium.

Our data show that the development of the typical biphasic population distribution was inhibited or delayed in cultures of strains mutant for HJ processing or double-strand break repair. A striking aspect of these results is that these distributions continued to develop throughout stationary phase, even up to 72 hr postinoculation. The *recG258 ruvA60* double-mutant strain, which is unable to process HJs, was the most defective in developing distinct peak 1 and peak 2 populations (Figure 6). HJs are an intermediate in the repair of double-strand breaks by the RecBCD pathway. That loss of the RecBCD enzyme, which initiates this pathway, had a phenotype similar to loss of HJ processing (Figure 7) strongly suggests that the repair of double-strand breaks in stationary phase is required for the development of the populations detected by cytometric analysis.

In our experiments, the effects of inactivating these repair pathways were manifested as cells entered stationary phase; however, the precise temporal requirement for their activities is not clear, and at least two scenarios are possible. The first scenario is that the activities of these proteins are required to repair the endogenous DNA damage that occurs during exponential growth to ensure that genome-processing events in stationary phase occur smoothly. The second scenario is that their activities are required for genome-processing events during stationary phase. Depending on when these repair proteins act in this genome-processing pathway, different models can be proposed to explain their roles.

One explanation for the requirement of these proteins for stationary-phase chromosome processing is that they are involved in ongoing repair of endogenous DNA damage that occurs during exponential growth to ensure that the chromosomes are free of damage *before* the cells enter stationary phase. This ongoing repair ensures that the chromosomes are intact and ready for efficient processing as cells enter stationary phase. Thus, in wild-type cultures, most cells enter the peak 1 population rapidly during stationary phase. In contrast, cells of the repair mutant strains could arrive at the onset of stationary phase with damaged chromosomes due to failed repair of DNA damage that occurred during exponential growth. This unrepaired damage could block or slow late-stage chromosome processing pathways (such as chromosome partitioning), causing the persistence of cell populations with higher DNA content during stationary phase. In support of this model, earlier studies by Ishioka *et al.* (1998, 1997) and more recent studies by Rudolph *et al.* (2009a, 2009b) have demonstrated that the RecG and the Ruv pathways are required to ensure proper chromosome segregation, at least in UV-irradiated and multiply repair-defective cells.

If the activities of these repair proteins are required *after* exponential growth as cells enter stationary phase, then at least three possible explanations can be proposed. One explanation is that cells continue to divide late into stationary phase, partitioning their DNA to daughter cells by a mechanism similar to, but slower than, that of exponentially growing cells. The energy required for this active process could come from the metabolism of nutrients released from dead cells in the culture. If this hypothesis is correct, then HJ processing and double-strand break repair would be implicated in chromosome partitioning, or perhaps decatenation, in late-stationary phase. Normal chromosomal decatenation after DNA replication is a function of the XerCD and FtsK proteins (Grainge *et al.* 2007), and the RuvABC, RecG, and RecBCD proteins have not previously been implicated in this process. However, cells in late-stationary phase might need HJ processing and double-strand break repair because of increased DNA damage from genotoxic agents that accumulate in the medium.

A second explanation is that the peaks detected by flow cytometry do not consist of populations of cells with different numbers of chromosomes or different amounts of DNA, but instead, consist of populations of cells with different degrees of DNA compaction. During stationary phase, chromosomes are compacted via their association with DNA binding proteins, which include HU (Sarkar *et al.* 2007), MukB (Cui *et al.* 2008), and Dps (Frenkiel-Krispin *et al.* 2004). This compaction may decrease the ability of dyes like propidium iodide to bind the DNA duplex, resulting in the decreased fluorescence detected during flow cytometric analysis. Previous studies using flow cytometry of *E. coli* cells have not considered this possibility (Åkerlund *et al.* 1995; Boye and Løbner-Olesen 1991). This second hypothesis is particularly interesting, as it proposes that RuvABC, RecG, and RecBCD are involved in chromosome remodeling during stationary phase.

Finally, a third and perhaps less likely explanation is that controlled DNA degradation occurs during late-stationary phase until the cells have uniform chromosome numbers. Such DNA degradation has not been reported previously, but it would explain the apparent reduction in DNA content (increase in numbers of cells with lower fluorescence intensity) that occurs in wild-type cells in the absence of cell division. Why HJ processing and double-strand break repair would be required for this process is not clear, but such a requirement would represent novel functions for these proteins. A converse possibility is that DNA replication, but not cell division, may continue longer into stationary phase in the mutant strains than in the wild-type strain, resulting in a higher proportion of cells with more DNA (increase in numbers of cells with higher fluorescence intensity); however, as shown in Table 2, we were unable to detect the DNA synthesis that would be required in the mutant strains if this hypothesis were true.

That DNA manipulation continues in late–stationary-phase cells is supported by several other observations. First, expression of *E. coli*’s three inducible, specialized DNA polymerases, DNA Pol II, Pol IV, and Pol V, continues for days after cells reach stationary phase (Yeiser *et al.* 2002). In addition, the error-prone Y-family Pol IV is upregulated in late–stationary-phase cells under control of the stationary-phase sigma factor, RpoS (Layton and Foster 2003; Storvik and Foster 2010). The reason for this late induction is not fully understood; however, Pol IV has been hypothesized to synthesize DNA during recombination-dependent DNA repair leading to adaptive Lac^+^ mutations (Foster 2000; Mckenzie *et al.* 2001; Tompkins *et al.* 2003). As mentioned above, the DNA polymerase processivity factor DnaN and the RecA-loading factor RecF are also induced in stationary phase (Villarroya *et al.* 1998), further supporting the idea that DNA repair and recombination occur even in the absence of bulk DNA replication and cell division.

This work has demonstrated the requirements of RuvABC, RecG, and RecBCD for establishment and/or maintenance of DNA content in stationary-phase cells. Determining whether double-strand break repair and HJ processing are required during exponential growth to “set the stage” during stationary phase for proper genome processing, for chromosome partitioning and/or reorganization, or for controlled DNA degradation will require further experimentation. For example, artificially inducing double-strand breaks at various points in the cell cycle might elucidate when double-strand break repair functions are required. Further research is needed to understand fully the molecular processes that allow nondividing cells to manage their DNA content and chromosome organization, and our data suggest that flow cytometry is a useful technique for such investigations.

## Supplementary Material

Supporting Information
