# Supplementary material for: Double-Strand Break Repair and Holliday Junction Processing Are Required for Chromosome Processing in Stationary-Phase Escherichia coli Cells
Source: G3 (Bethesda). 2011 Nov 1;1(6):417–26. doi: 10.1534/g3.111.001057 (PMC3276156; doi:10.1534/g3.111.001057)
Supplement: Supporting Information [file supp_1_6_417__index.html]

Supporting Information 

# Double-Strand Break Repair and Holliday Junction Processing Are Required for Chromosome Processing in Stationary-Phase *Escherichia coli* Cells

## Supporting Information for Williams, Hetrick, and Foster, 2011

**Files in this Data Supplement:**

- Supporting Information - Figures S1-S6 (PDF, 2.2 MB)
- Figure S1 - The distribution of cells with distinct fluorescence intensities is stable in late stationary phase cultures (PDF, 384 KB)
- Figure S2 - Loss of RecG helicase has only minor effects on the distribution of populations with distinct fluorescence intensities in late stationary phase cultures (PDF, 320 KB)
- Figure S3 - The RuvABC complex is required for normal fluorescence population dynamics in late stationary phase cultures (PDF, 304 KB)
- Figure S4 - Loss of both Holliday junction-processing pathways severely alters fluorescent population dynamics in late stationary phase cultures (PDF, 312 KB)
- Figure S5 - Loss of RecB has only minor effects on the fluorescent population dynamics in late stationary phase cultures (PDF, 328 KB)
- Figure S6 - EdU labeling to measure ongoing DNA replication (PDF, 716 KB)
