# Supplementary material for: Double-Strand Break Repair and Holliday Junction Processing Are Required for Chromosome Processing in Stationary-Phase Escherichia coli Cells
Source: G3 (Bethesda). 2011 Nov 1;1(6):417–26. doi: 10.1534/g3.111.001057 (PMC3276156; doi:10.1534/g3.111.001057)
Supplement: Supporting Information [file supp_1.6.417_001057SI.pdf]

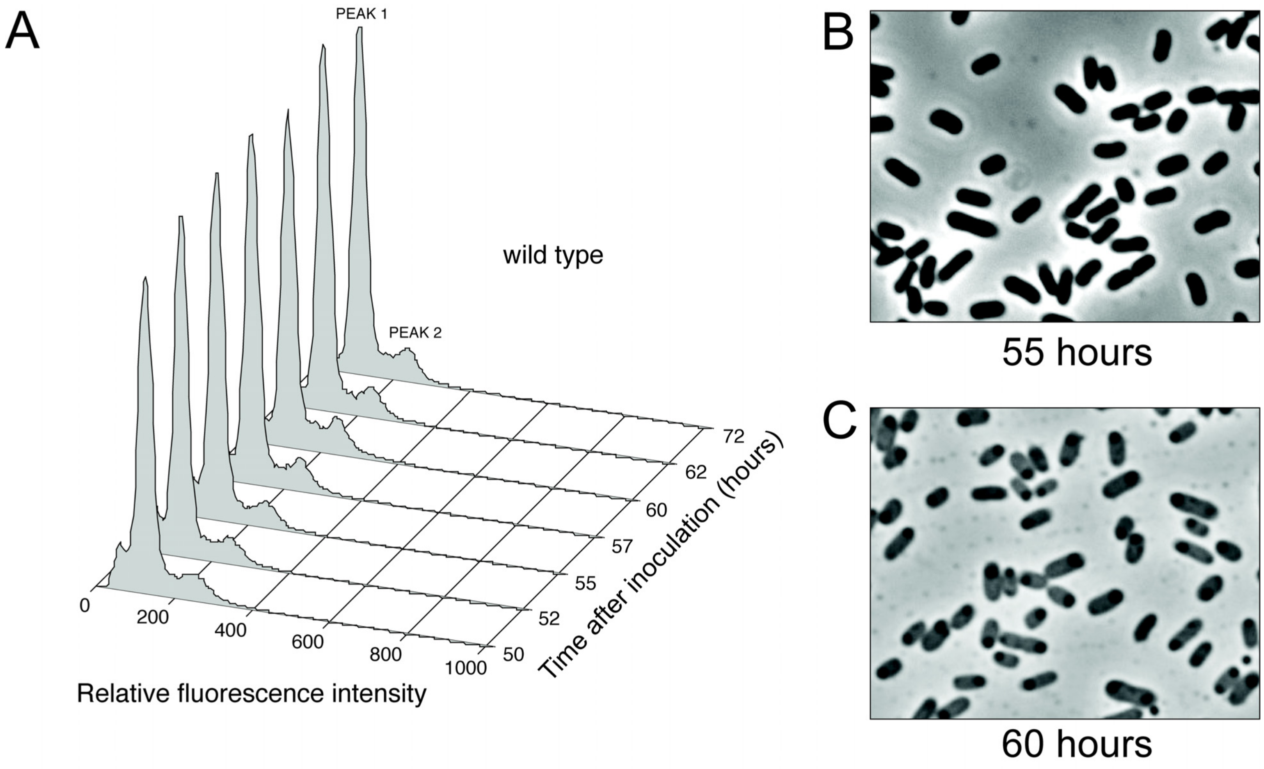

Figure S1 The distribution of cells with distinct fluorescence intensities is stable in late stationary phase cultures. Propidium iodide stained cells (strain FC36) were analyzed by flow cytometry at the indicated time points. (A) The numbers of cells (y-axis) are plotted against their relative fluorescence intensities (x-axis). The histograms from the time points indicated are aligned along the z-axis. (B and C) Phase-contrast micrographs of unfixed cells from the time points indicated (1000X magnification).

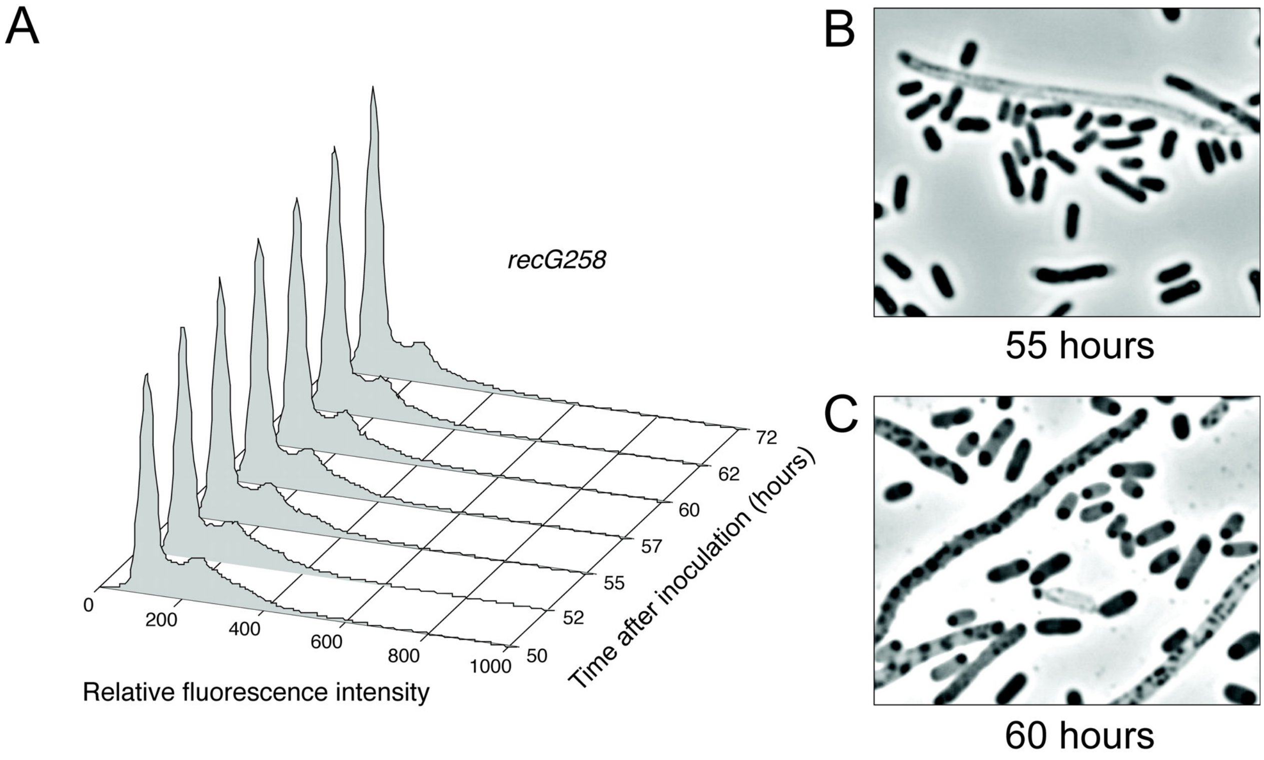

Figure S2 Loss of RecG helicase has only minor effects on the distribution of populations with distinct fluorescence intensities in late stationary phase cultures. Propidium iodide stained cells (strain FC457) were analyzed by flow cytometry at the indicated time points. (A) The numbers of cells (y-axis) are plotted against their relative fluorescence intensities (x-axis). The histograms from the time points indicated are aligned along the z-axis. (B and C) Phase-contrast micrographs of unfixed cells from the time points indicated (1000X magnification).

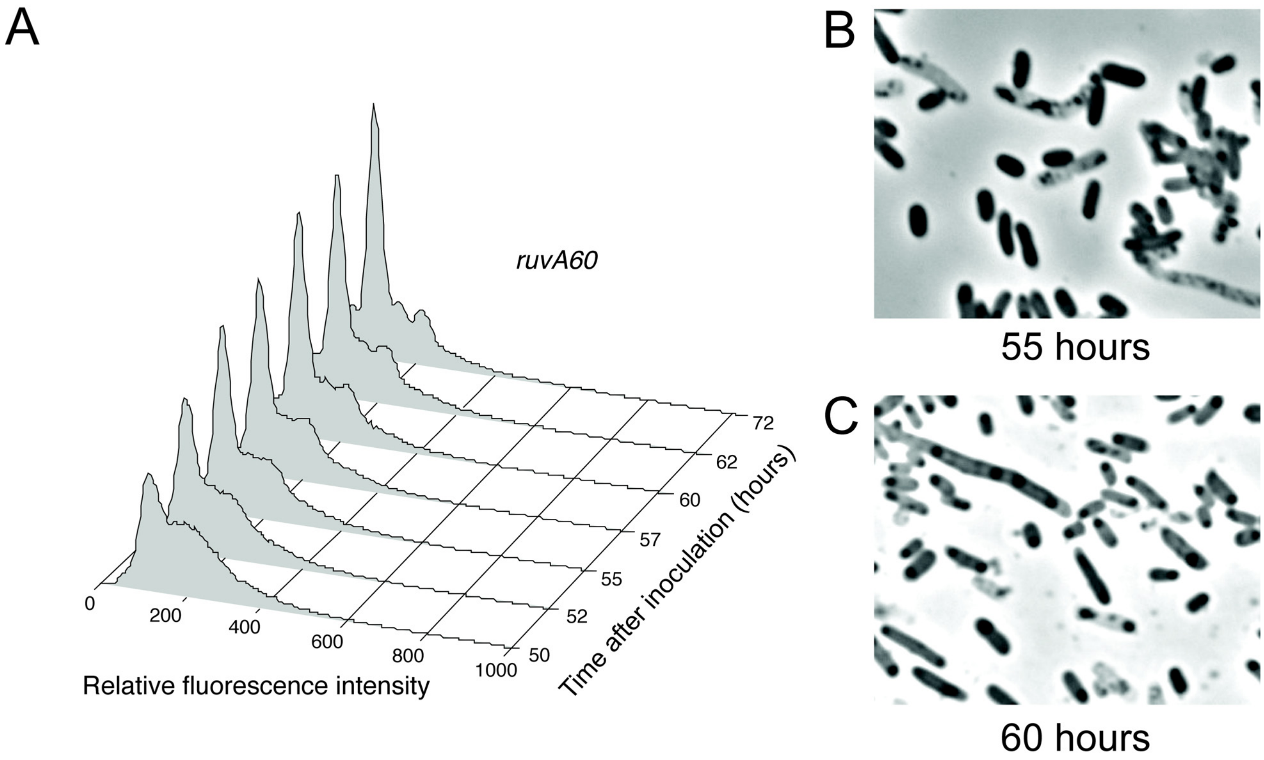

Figure S3 The RuvABC complex is required for normal fluorescence population dynamics in late stationary phase cultures. Propidium iodide stained cells (strain FC484) were analyzed by flow cytometry at the indicated time points. (A) The numbers of cells (y-axis) are plotted against their relative fluorescence intensities (x-axis). The histograms from the time points indicated are aligned along the z-axis. (B and C) Phase-contrast micrographs of unfixed cells from the time points indicated (1000X magnification).

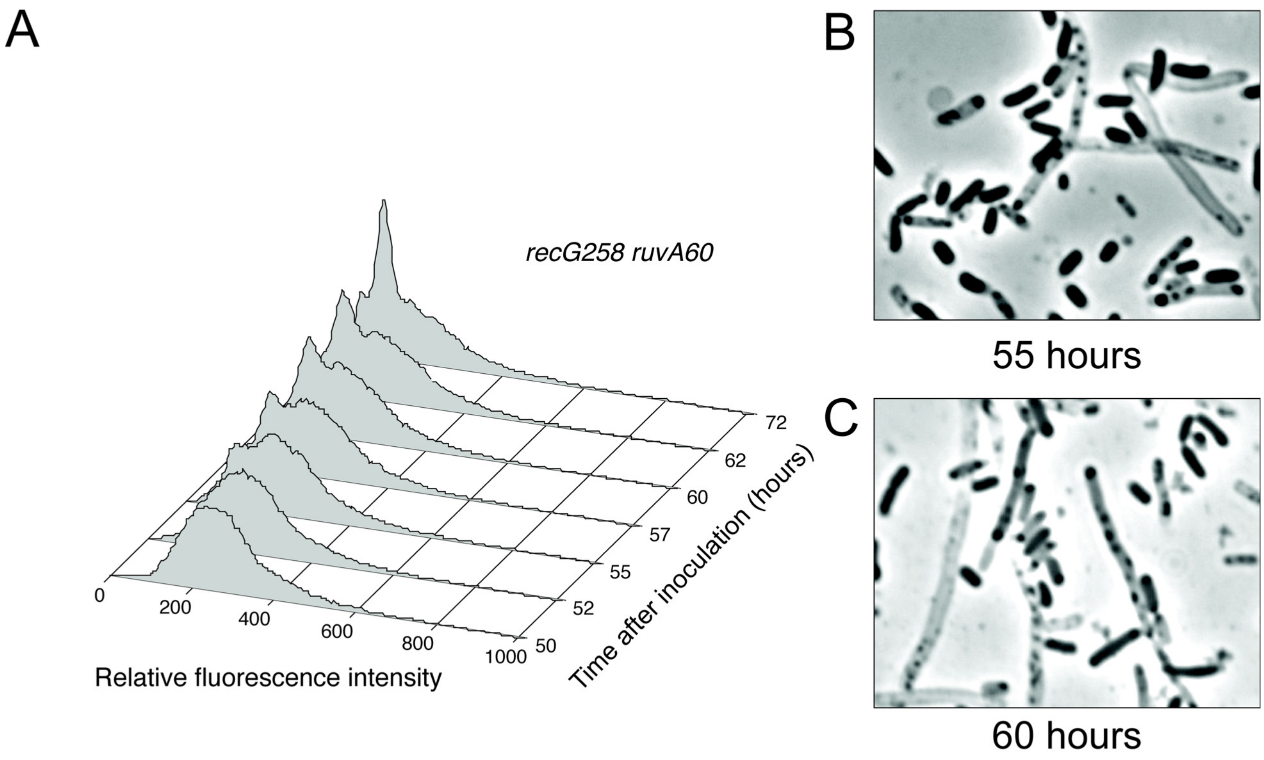

Figure S4 Loss of both Holliday junction-processing pathways severely alters fluorescent population dynamics in late stationary phase cultures. Propidium iodide stained cells (strain FC513) were analyzed by flow cytometry at the indicated time points. (A) The numbers of cells (y-axis) are plotted against their relative fluorescence intensities (x-axis). The histograms from the time points indicated are aligned along the z-axis. (B and C) Phase-contrast micrographs of unfixed cells from the time points indicated (1000X magnification).

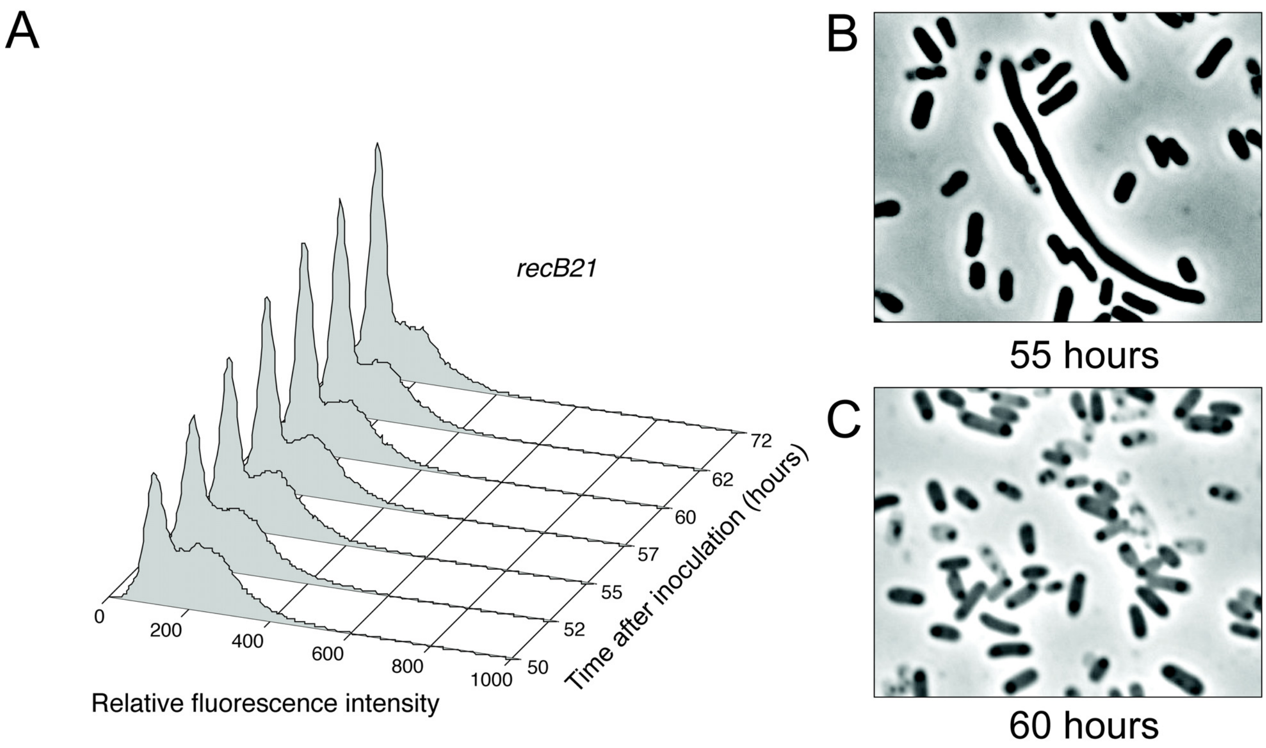

Figure S5 Loss of RecB has only minor effects on the fluorescent population dynamics in late stationary phase cultures. Propidium iodide stained cells (strain FC400) were analyzed by flow cytometry at the indicated time points. (A) The numbers of cells (y-axis) are plotted against their relative fluorescence intensities (x-axis). The histograms from the time points indicated are aligned along the z-axis. (B and C) Phase-contrast micrographs of unfixed cells from the time points indicated (1000X magnification).

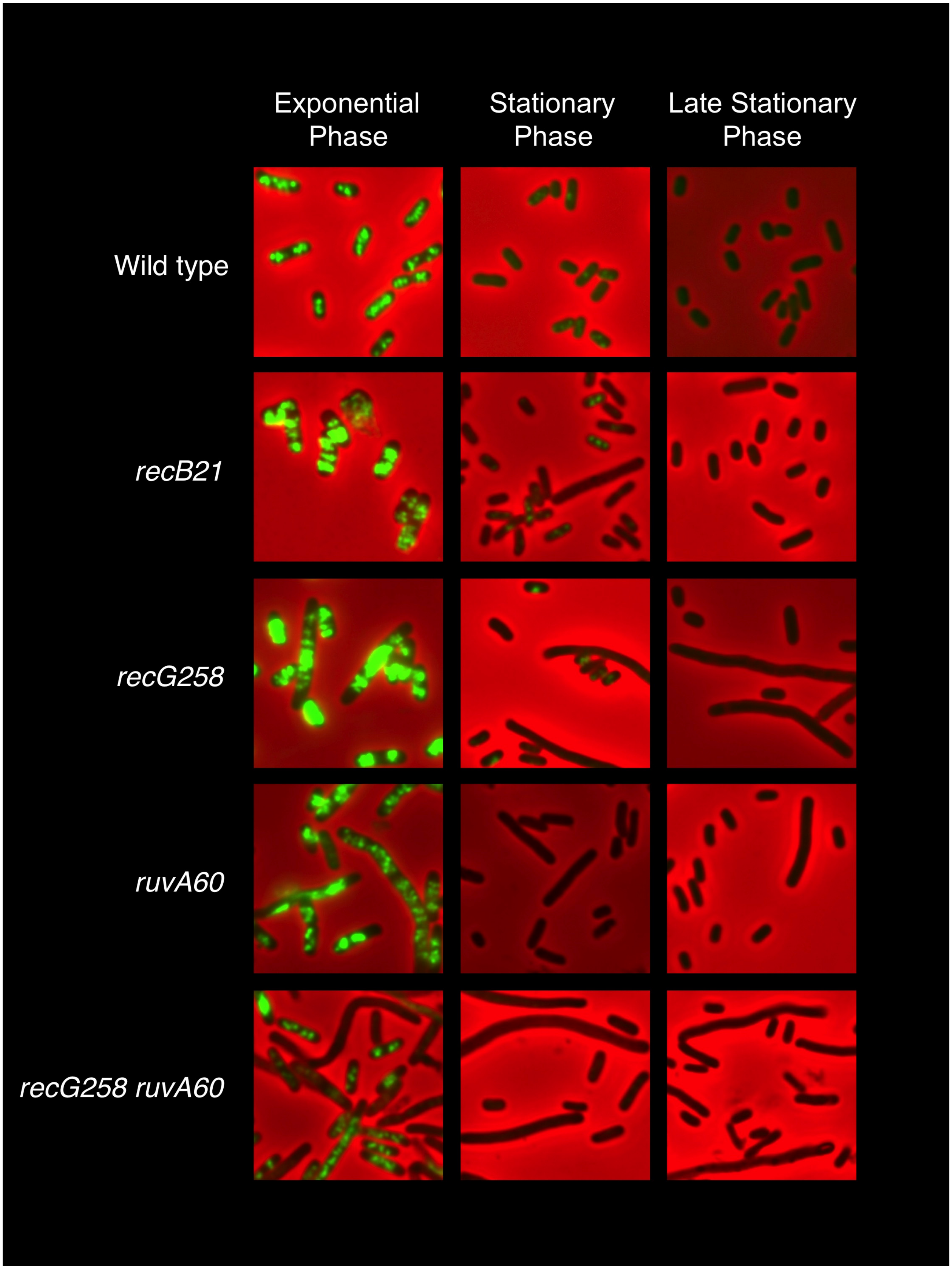
Exponential Phase
Stationary Phase
Late Stationary Phase
Wild type
recB21
recG258
ruvA60
recG258 ruvA60

Figure S6 EdU labeling to measure ongoing DNA replication. Representative combined phase-contrast and fluorescence images of EdU-labeled cells in exponential phase, stationary phase, and late stationary phase are shown. Cultures were grown, exposed to EdU, harvested, and visualized as described in Materials and Methods. Representative fields of view are shown. For each field, the time of exposure for the phase-contrast image was adjusted for maximal contrast, but all fluorescence image exposure times were 500 milliseconds. The phase-contrast image and fluorescence image for each field were combined and false-colored red and green, respectively, with Metamorph image software. Wild type = FC36; *recB21*= FC400; *recG258* = FC457; *ruvA60* = FC484; *recG258 ruvA60* = FC513.
