# Supplementary material for: Double-Strand Break Repair and Holliday Junction Processing Are Required for Chromosome Processing in Stationary-Phase Escherichia coli Cells
Source: G3 (Bethesda). 2011 Nov 1;1(6):417–26. doi: 10.1534/g3.111.001057 (PMC3276156; doi:10.1534/g3.111.001057)
Supplement: Supporting Information [file supp_1.6.417_FigureS1.pdf]

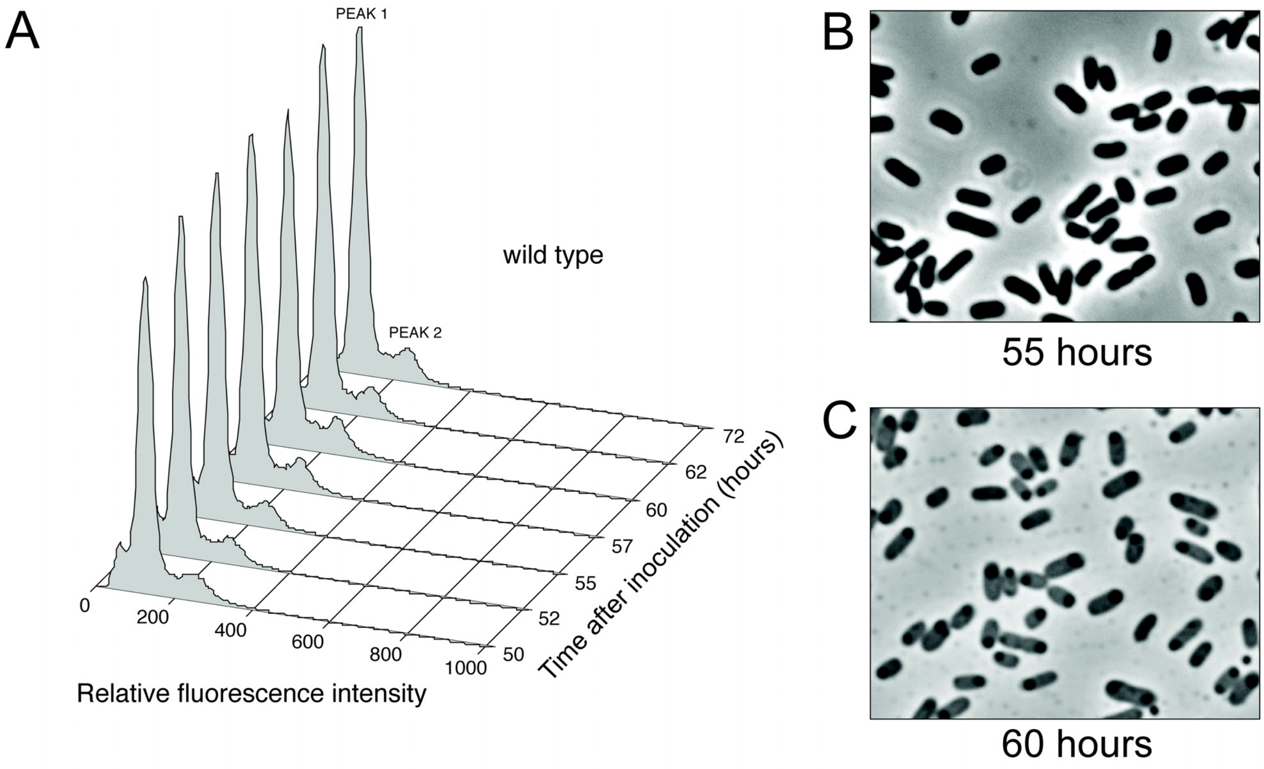

Figure S1 The distribution of cells with distinct fluorescence intensities is stable in late stationary phase cultures. Propidium iodide stained cells (strain FC36) were analyzed by flow cytometry at the indicated time points. (A) The numbers of cells (y-axis) are plotted against their relative fluorescence intensities (x-axis). The histograms from the time points indicated are aligned along the z-axis. (B and C) Phase-contrast micrographs of unfixed cells from the time points indicated (1000X magnification).
