# Supplementary material for: Double-Strand Break Repair and Holliday Junction Processing Are Required for Chromosome Processing in Stationary-Phase Escherichia coli Cells
Source: G3 (Bethesda). 2011 Nov 1;1(6):417–26. doi: 10.1534/g3.111.001057 (PMC3276156; doi:10.1534/g3.111.001057)
Supplement: Supporting Information [file supp_1.6.417_FigureS6.pdf]

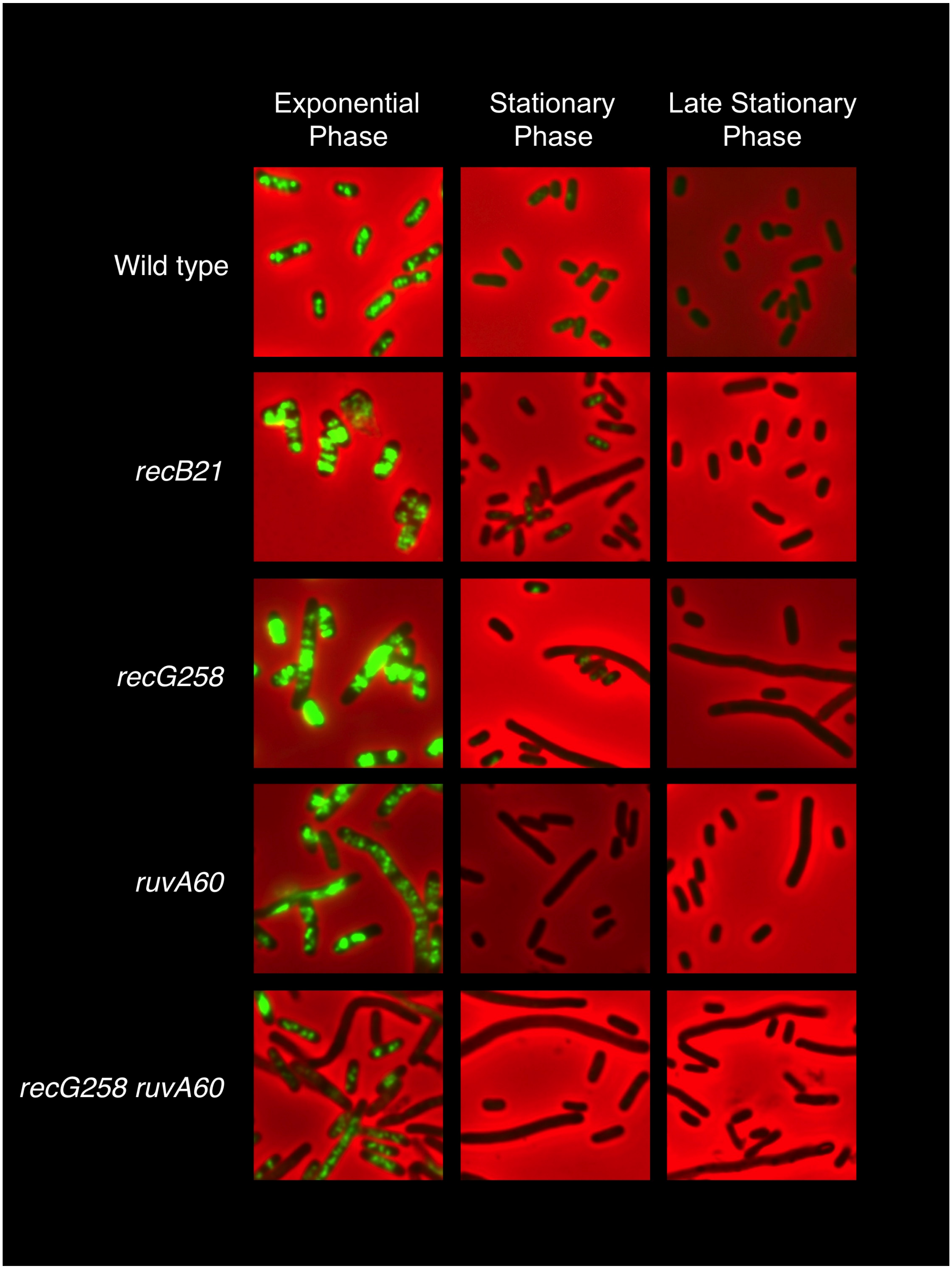
Exponential Phase
Stationary Phase
Late Stationary Phase
Wild type
recB21
recG258
ruvA60
recG258 ruvA60

Figure S6 EdU labeling to measure ongoing DNA replication. Representative combined phase-contrast and fluorescence images of EdU-labeled cells in exponential phase, stationary phase, and late stationary phase are shown. Cultures were grown, exposed to EdU, harvested, and visualized as described in Materials and Methods. Representative fields of view are shown. For each field, the time of exposure for the phase-contrast image was adjusted for maximal contrast, but all fluorescence image exposure times were 500 milliseconds. The phase-contrast image and fluorescence image for each field were combined and false-colored red and green, respectively, with Metamorph image software. Wild type = FC36; *recB21*= FC400; *recG258* = FC457; *ruvA60* = FC484; *recG258 ruvA60* = FC513.
